# Effect of the CYP3A inhibitors, diltiazem and ketoconazole, on ticagrelor pharmacokinetics in healthy volunteers

**DOI:** 10.3109/21556660.2013.785413

**Published:** 2013-03-15

**Authors:** Renli Teng, Kathleen Butler

**Affiliations:** AstraZeneca LP, Wilmington, DelawareUSA

**Keywords:** Antiplatelet therapy, Diltiazem, Ketoconazole, Pharmacokinetics, Ticagrelor

## Abstract

**Objectives:**

Two open-label, two-period, crossover studies in healthy volunteers were designed to determine the pharmacokinetic interactions between ticagrelor, a P2Y_12_ receptor antagonist, and a moderate (diltiazem) and a strong (ketoconazole) cytochrome P450 (CYP) 3A inhibitor.

**Methods:**

Seventeen volunteers received diltiazem (240 mg once daily) for 14 days. In the second study, ketoconazole (*n* = 14) 200 mg twice daily was given for 10 days. A single oral 90-mg ticagrelor dose was administered on day 8 (diltiazem) or day 4 (ketoconazole). In each study, volunteers received a single 90-mg oral dose of ticagrelor before or after washout (≥14 days). Pharmacokinetic parameters for ticagrelor, AR-C124910XX (primary metabolite), diltiazem, and ketoconazole were assessed.

**Results:**

Compared with ticagrelor alone, diltiazem co-administration significantly increased the mean maximum concentration (*C*_max_) and mean area under the plasma concentration–time curve (AUC) for ticagrelor by 69% and 174%, respectively. Diltiazem co-administration reduced *C*_max_ by 38% but had no significant effect on AUC for AR-C124910XX. *C*_max_ and AUC for ticagrelor were increased by 135% and 632%, respectively, by ketoconazole co-administration, whereas these parameters were reduced by 89% and 56%, respectively, for AR-C124910XX. Diltiazem and ketoconazole pharmacokinetic parameters were not significantly affected by the presence of ticagrelor.

**Conclusions:**

These results suggest that ticagrelor can be co-administered with moderate CYP3A inhibitors. However, co-administration of strong CYP3A inhibitors with ticagrelor is not recommended.

## Introduction

Ticagrelor is an oral P2Y_12_ receptor antagonist that inhibits adenosine diphosphate-induced platelet aggregation1. Results from the PLATelet inhibition and patient Outcomes (PLATO) phase III trial showed that, compared with clopidogrel + aspirin, ticagrelor + aspirin significantly reduced the rate of myocardial infarction/stroke/death from vascular causes in patients with acute coronary syndromes (ACS)2. Subsequently, ticagrelor has been approved for use in the prevention of atherothrombotic events in adult ACS patients in more than 70 countries, including the EU3 and the United States4. Recently updated European guidelines recommend ticagrelor combined with aspirin as one of several antiplatelet therapies for managing ACS patients with non-ST-segment elevation5. Furthermore, updated European guidelines also gave ticagrelor a Class I recommendation for patients presenting with persistent ST-segment elevation6.

The complexity of ACS necessitates the use of multiple drugs to manage the disease and a wide variety of co-morbidities7,8. Consequently, drug–drug interactions may occur which could result in altered exposure to a co-administered drug, thereby affecting efficacy and safety9,10. The most abundant drug-metabolizing enzyme in the liver is cytochrome P450 (CYP) 3A11,12, and clinically important interactions involving this enzyme are well documented12. Although ticagrelor is a direct-acting antiplatelet agent, it is metabolized to at least ten metabolites13. The major metabolite, AR-C124910XX, which is present at approximately 30–40% of the levels of ticagrelor13–17, is approximately equipotent in inhibiting platelet aggregation (AstraZeneca, data on file). Experiments with human liver microsomes demonstrated that ticagrelor is principally metabolized by CYP3A18.

AR-C124910XX is further metabolized by UDP-glucuronosyltransferase to a highly polar glucuronidated metabolite, or via hydroxylation by an unknown isoenzyme to a minor hydroxylated derivative. Given that the glucuronidated metabolite is highly polar and expected to be excreted rapidly in the urine, and the hydroxylated metabolite is minor, the pharmacological relevance of these biotransformations is likely to be minimal13.

The evaluation of potential drug–drug interactions is important in drug development. Known inhibitors of CYP3A are commonly used as model compounds to investigate such interactions10. Diltiazem is a calcium channel blocker used to treat angina pectoris and mild-to-moderate arterial hypertension19. This compound is an inhibitor of CYP3A activity20,21 and is classified as a moderate inhibitor of this enzyme10. The antifungal agent, ketoconazole22,23, also inhibits CYP3A24 and is considered to be a strong CYP3A inhibitor10. Both of these agents are commonly used in evaluating the effects of CYP3A inhibition on the pharmacokinetic parameters of co-administered drugs10,21,25.

Given the key role of CYP3A in drug metabolism, including that of ticagrelor, and multidrug use in ACS, two drug–drug interaction studies in healthy volunteers were conducted. The primary objectives of these studies were to assess the effects of co-administration of a moderate (diltiazem) and a strong (ketoconazole) CYP3A inhibitor on ticagrelor pharmacokinetic parameters. Secondary objectives included: assessment of AR-C124910XX pharmacokinetic parameters; effect of ticagrelor on pharmacokinetic parameters of diltiazem and ketoconazole; safety and tolerability.

## Patients and methods

### Study populations

Key inclusion criteria for both studies were: males or females (post-menopausal or surgically sterile); age 18–45 years; body weight ≥50 kg; and a body mass index (BMI) of 18–30 kg/m^2^. For both studies, the key exclusion criteria included: smoking/tobacco use in the past 6 months; use of drugs known to increase propensity for bleeding within 2 weeks of study start; a history or presence of any condition known to interfere with drug absorption, distribution, metabolism, or excretion; alcohol or substance abuse in the previous 12 months; consumption of Seville oranges, grapefruit-containing products, alcohol, medicines, or nutritional supplements within 1 week of study start.

For both studies, all volunteers provided informed consent in writing. The protocols were approved by an institutional review board (diltiazem study: Southern Institutional Review Board, Miami, FL, USA; ketoconazole study: Research Consultants’ Review Committee, West Austin, TX, USA). The studies were conducted according to AstraZeneca bioethics policy, applicable regulatory requirements, and in accordance with the Declaration of Helsinki and in line with good clinical practice.

### Study designs and treatment

Both studies were single-center, randomized, open-label studies with a two-period, in-patient, crossover design. For both studies, a low non-loading dose of ticagrelor (i.e., a single oral dose of 90 mg) was selected, so if an interaction occurred between ticagrelor and the CYP3A4 inhibitor, then the increase in ticagrelor plasma concentrations would minimize any safety issues. The maximum tolerated single dose of ticagrelor is 900 mg26,27. The approved dosing regimen of ticagrelor is 180 mg loading dose followed by 90 mg twice daily thereafter3,4. The daily dose (240 mg) of diltiazem selected for the ticagrelor-diltiazem study was within the approved clinical range (180–480 mg daily19), and was expected to inhibit CYP3A4^21^. The selected dose of ketoconazole (200 mg twice daily) is the maximum clinically approved oral dose23 and is commonly used in phase I drug–drug interaction studies10,25 to inhibit CYP3A4 activity.

**Figure 1.**
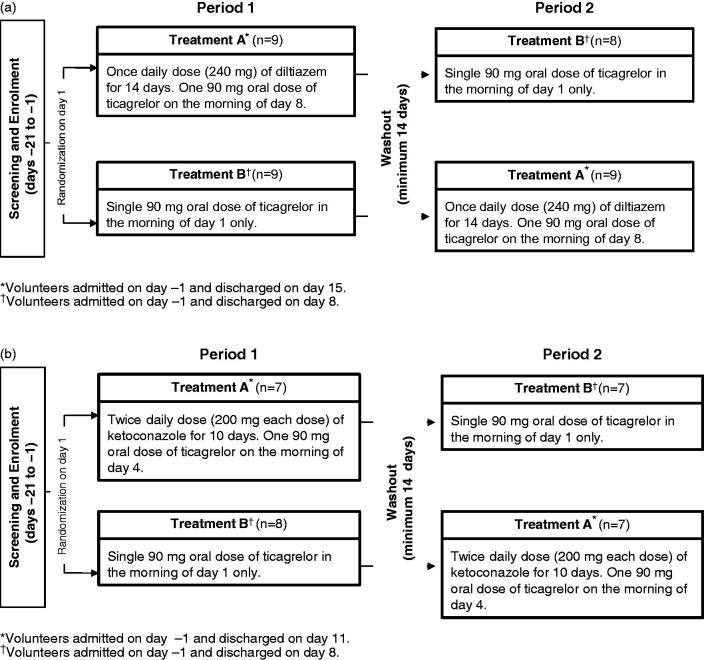
Study design. (a). Diltiazem study (*n* = 17). (b). Ketokonazole study (*n* = 14).

Figure 1 depicts the overall study design for both studies. At the start of each study, volunteers were randomized to one of two treatments (A or B). After the washout period, volunteers received the alternate dosing regimen. On the scheduled day, ticagrelor was co-administered with the CYP3A4 inhibitor in the morning after fasting for ≥10 hours followed by a further fast for ≥4 hours. Water was permitted *ad libitum* throughout each period except for 2 hours before and after ticagrelor administration.

For each study, the volunteers were restricted from consuming alcohol, caffeine-containing products, Seville oranges or grapefruit-containing foods, over-the-counter preparations (including herbal remedies), and any drug known to increase bleeding.

### Pharmacokinetic sample collection

In both studies, blood samples (2 or 3 mL) were collected for the evaluation of ticagrelor and AR-C124910XX plasma concentrations at the following times: 0, 0.5, 1, 2, 3, 4, 6, 8, 10, 12, 16, 20, 24, 36, 48, 72, 96, 120, 144, and 168 hours post-ticagrelor dosing on day 1 (i.e., following ticagrelor alone in both studies), day 4 (i.e., after ticagrelor co-administration with ketoconazole), and day 8 (i.e., after ticagrelor co-administration with diltiazem). The exceptions to these sampling times, in the ticagrelor-ketoconazole study, were: on days 1 and 4 when a sample was collected at 18 hours (instead of at 16 and 20 hours) post-ticagrelor dosing; on day 4 additional samples were collected at 60, 84, 108, 132, and 156 hours post-ticagrelor dosing.

Blood samples for analysis of diltiazem were collected pre-dose on days 1–6 and 10–14 for analysis of trough concentrations, and at 0, 0.5, 1, 2, 3, 4, 6, 8, 10, 12, 16, 20, and 24 hours post-diltiazem dosing on days 7 and 8. For analysis of ketoconazole, blood samples were collected pre-dosing on days 3 and 4, and at 12 hours post-ketoconazole dosing on day 4.

All plasma samples were stored at −20°C until assayed.

### Analytical methods

Ticagrelor, AR-C124910XX, diltiazem, and ketoconazole were analyzed using fully-validated, reversed-phase liquid chromatography with tandem mass spectrometry methods. The limits of quantification (LOQ) for ticagrelor and AR-C124910XX were 5 and 2.5 ng/L, respectively28. The LOQ for diltiazem was 1 ng/mL, and 10 ng/mL for ketoconazole (AstraZeneca, data on file).

### Data analyses

In a previous healthy volunteer study (*n* = 8) with a single oral dose of ticagrelor (100 mg; D5130C5266, AstraZeneca data on file), the inter-subject coefficients of variation (CV) were 40% for the maximum plasma concentration (*C*_max_) and 42% for the area under the plasma concentration–time curve (AUC). Based on this variability and assuming an intra-subject correlation of 0.65, a two one-sided testing procedure (α-level = 0.05; true ratio = 1) estimated that a sample size of 12 healthy volunteers would provide a statistical power of 90% for the 90% confidence intervals (CI) for *C*_max_ and AUC to be contained within a pre-specified no-effect range of 0.70–1.43 for the ticagrelor/ketoconazole study. Using the variability data observed in the ticagrelor/ketoconazole study (CV 38.5% for *C*_max_ and 38.9% for AUC) and the same assumptions, power requirements, and no-effect range, the sample size for the ticagrelor/diltiazem study was also estimated to be 12 healthy volunteers.

Pharmacokinetic parameters for ticagrelor, AR-C124910XX, and diltiazem were estimated by standard non-compartmental analyses (WinNonlin Professional, Pharsight Corporation, Mountain View, California, USA). Plasma concentration–time data were used to calculate *C*_max_, time to maximum concentration (*t*_max_), AUC, and the terminal elimination half-life (*t*_½_). The latter was calculated as 0.693/λz, where λz is the terminal phase elimination rate constant, derived by least-squares regression analysis of the plasma concentration–time data obtained over the terminal log-linear phase. AUC was calculated using the linear trapezoidal method and extrapolated to infinity. AR-C124910XX:ticagrelor ratios for *C*_max_ and AUC were calculated. Diltiazem pharmacokinetic parameters were estimated based on steady-state concentrations. Ketoconazole steady-state plasma concentrations were expressed as mean, CV, and range.

Statistical analyses were conducted using SAS version 8 (SAS Institute, Cary, North Carolina, USA). Pharmacokinetic parameters were summarized by descriptive statistics. AUC and *C*_max_ values for ticagrelor and AR-C124910XX were analyzed, following log-transformation, by analysis of variance with terms for treatment, period, and sequence, and the volunteer within sequence was included as a random effect. After exponentiation, geometric least square mean point estimates and 90% CIs for the ratio ticagrelor + CYP3A4 inhibitor/ticagrelor alone were calculated. The pre-specified limits for the 90% CI values indicating no interaction between ticagrelor and either CYP3A4 inhibitor were 0.7–1.43. For the potential effect of ticagrelor on diltiazem parameters, the pre-specified limits for 90% CI were 0.8–1.25.

### Safety and tolerability

Safety and tolerability of ticagrelor alone and in the presence of diltiazem or ketoconazole were evaluated by assessment of adverse events (AEs), vital signs, electrocardiographic (ECG), physical examination, and laboratory parameters (clinical chemistry, hematology, and urinalysis) throughout the studies.

## Results

### Baseline demographics and characteristics, and disposition

Eighteen volunteers were enrolled in the diltiazem study to ensure that at least 12 volunteers were evaluable. The majority were male (14/18, 78%), and all were Hispanic. The mean (range) age was 33 (18–44) years, and the mean (range) BMI was 27 (22–30) kg/m^2^. In the ketoconazole study, 16 volunteers were randomized to ensure that at least 12 volunteers were evaluable. Thirteen (81%) were male, and the majority (12/16, 75%) were Caucasian. The mean (range) age was 30 (20–45) years, and the mean (range) BMI was 25 (19–31) kg/m^2^.

One volunteer in the diltiazem study was withdrawn from the study at the start of the second period due to a positive drug screen. In the ketoconazole study, two volunteers discontinued the study. One volunteer was withdrawn following ticagrelor + ketoconazole administration in the first period due to a right bundle branch block. The other volunteer was withdrawn during ticagrelor + ketoconazole administration in the second period due to non-compliance.

### Effect of diltiazem on ticagrelor pharmacokinetic parameters

**Figure 2.**
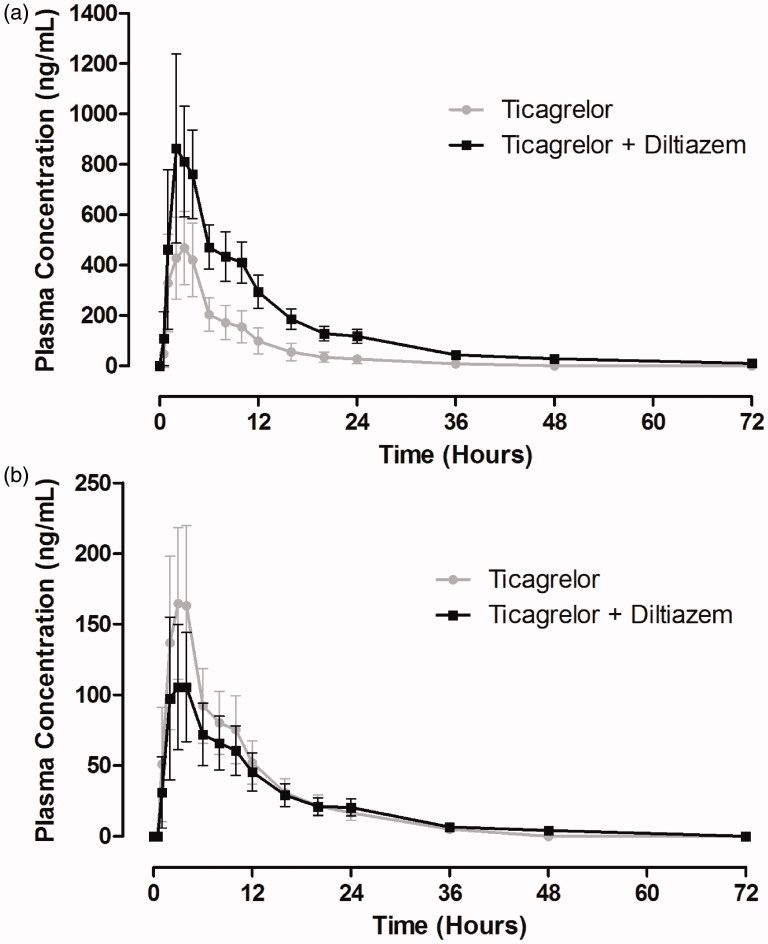
Mean (± standard deviation) plasma concentration–time profiles of ticagrelor (a) and AR-C124910XX (b) following a single 90-mg oral dose of ticagrelor in the presence and absence of diltiazem (240 mg once daily) (*n* = 17).

Co-administration of diltiazem with ticagrelor increased plasma concentrations of ticagrelor (Figure 2a). Table 1 shows the pharmacokinetic parameters of ticagrelor in the presence and absence of diltiazem. Ticagrelor was rapidly absorbed; the median *t*_max_ was 2 hours following administration of ticagrelor alone, which was unaffected by diltiazem. The half-life of ticagrelor was increased by 96% by co-administration of diltiazem. For ticagrelor, *C*_max_ was increased by 69% and AUC by 174% by the presence of diltiazem. The 90% CIs of the geometric least-square (GLS) mean ratios for both *C*_max_ and AUC for ticagrelor + diltiazem/ticagrelor were outside the prespecified no-effect limits of 0.70–1.43 (Table 1).

**Table 1.** Pharmacokinetic parameters and statistical analyses after a single oral 90-mg dose of ticagrelor in the presence and absence of diltiazem.

Parameter*	Ticagrelor (90 mg) alone *n* = 17	Ticagrelor (90 mg) plus diltiazem (240 mg qd) *n* = 17	GLS mean ratio: point estimate (90% CI)
Ticagrelor
*C*_max_ (ng/mL)	519 (34)	878 (30)	1.69 (1.47–1.95)
AUC (ng·h/mL)	3701 (37)	10,099 (24)	2.74 (2.40–3.13)
*t*_½_ (h)	8.3 (2.3)	16.3 (4.4)	–
*t*_max_ (h)	2.0 (1.0–4.0)	2.0 (2.0–4.0)	–
AR-C124910XX
*C*_max_ (ng/mL)	173 (38)	109 (36)	0.62 (0.57–0.68)
AUC (ng·h/mL)	1630 (29)	1424 (29)	0.87 (0.83–0.92)
*t*_½_ (h)	8.4 (1.3)	12.5 (4.7)	–
*t*_max_ (h)	3.0 (2.0–4.0)	3.0 (2.0–4.0)	–
Metabolite:parent ratios
*C*_max_ ratio	0.3 (36)	0.1 (24)	–
AUC ratio	0.4 (39)	0.1 (28)	–

*Values are: geometric mean (% CV) for *C*_max_, AUC and metabolite:parent ratios; mean (standard deviation) for *t*_½_; median (range) for *t*_max_. qd, once daily; GLS, geometric least-square; CI, confidence interval; *C*_max_, maximum plasma concentration; AUC, area under the plasma concentration–time curve from time 0 to infinity; *t*_½_, terminal elimination half-life; *t*_max_, time to *C*_max_.

Plasma concentrations of AR-C124910XX were decreased following administration of ticagrelor with diltiazem compared with ticagrelor alone (Figure 2b). Pharmacokinetic parameters for AR-C124910XX are summarized in Table 1. This metabolite was rapidly formed; the median *t*_max_ was 3 hours after ticagrelor alone, which was unaffected by diltiazem. The half-life of AR-C124910XX was increased by 49% by co-administration of diltiazem with ticagrelor. The *C*_max_ for AR-C124910XX was decreased by 38% by diltiazem and the 90% CIs of the GLS mean ratio for ticagrelor + diltiazem/ticagrelor were outside the limits of 0.70–1.43. The AUC was slightly reduced (13%) by the co-administration of diltiazem, although the 90% CIs of the GLS mean ratio were within the prespecified limits (Table 1). The metabolite:parent ratios were decreased by 66% and 75% for *C*_max_ and AUC, respectively, by the presence of diltiazem (Table 1).

### Effect of ticagrelor on diltiazem pharmacokinetic parameters

Co-administration of ticagrelor had no effect on the pharmacokinetic parameters of diltiazem. At steady-state diltiazem, the mean (% CV) *C*_max_ and AUC were 194 (41) ng/mL and 2998 (41) ng·h/mL, respectively. In the presence of ticagrelor, *C*_max_ and AUC were 198 (47) ng/mL and 2880 (53) ng·h/mL, respectively. The point estimates (90% CIs) of the GLS mean ratios were 1.02 (0.89–1.17) for *C*_max_ and 0.96 (0.87–1.06) for AUC, which were both within the prespecified limits of 0.80–1.25. Median *t*_max_ (range) of diltiazem was not greatly affected by co-administration of ticagrelor, i.e. 8 (0–16) hours (diltiazem alone) and 10 (4–16) hours (diltiazem + ticagrelor).

### Effect of ketoconazole on ticagrelor pharmacokinetic parameters

**Figure 3.**
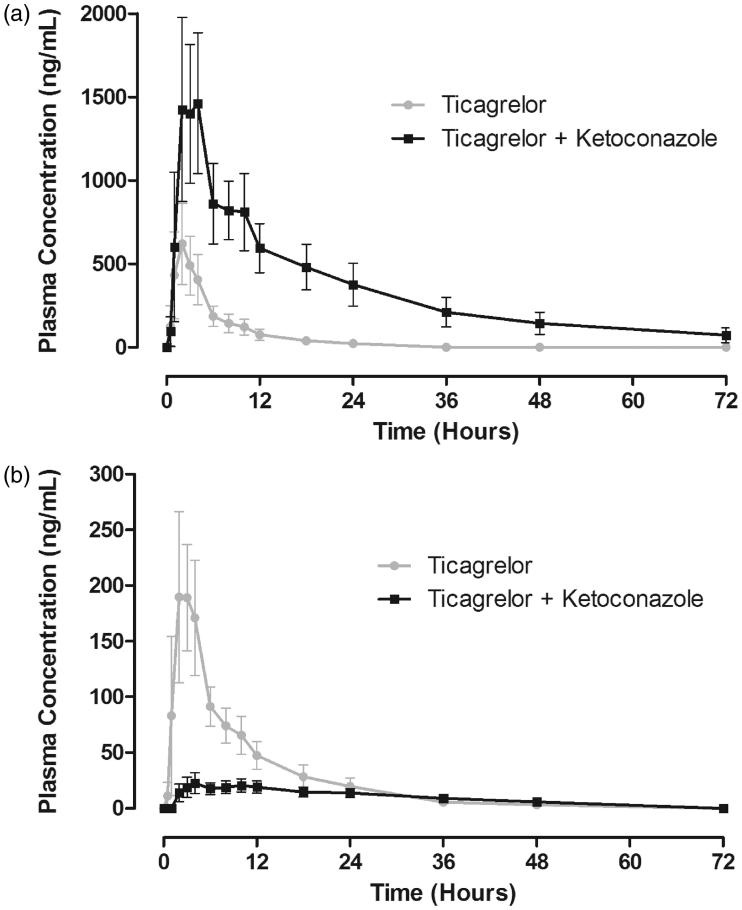
Mean (± standard deviation) plasma concentration–time profiles of ticagrelor (a) and AR-C124910XX (b) following a single 90-mg oral dose of ticagrelor in the presence and absence of ketoconazole (200 mg twice daily) (*n* = 17).

The plasma concentration–time profile of ticagrelor co-administered with ketoconazole was markedly increased compared with ticagrelor alone (Figure 3a). The median *t*_max_ of ticagrelor was similar (2 hours) in the presence and absence of ketoconazole (Table 2). The half-life of ticagrelor was increased to approximately 362% in the presence of ketoconazole. The *C*_max_ and AUC for ticagrelor were increased by 135% and 632%, respectively, by the co-administration of ketoconazole. The 90% CIs of the GLS mean ratio for both parameters were above the upper boundary of the limits of 0.70–1.43 (Table 2).

**Table 2.** Pharmacokinetic parameters and statistical analyses after a single oral 90-mg dose of ticagrelor in the presence and absence of ketoconazole (200 mg twice daily).

Parameter*	Ticagrelor (90 mg) alone *n* = 14	Ticagrelor (90 mg) plus ketoconazole (200 mg bid) *n* = 14	GLS mean ratio: point estimate (90% CI)
Ticagrelor
*C*_max_ (ng/mL)	654 (33)	1537 (30)	2.35 (2.13–2.60)
AUC (ng·h/mL)	3640 (35)	26,640 (36)	7.32 (6.43–8.34)
*t*_½_ (h)	7.1 (4.9–9.8)	25.7 (16.6–31.9)	–
*t*_max_ (h)	2.0 (1.0–3.0)	2.0 (2.0–4.1)	–
AR-C124910XX
*C*_max_ (ng/mL)	207 (29)	23 (33)	0.11 (0.09–0.14)
AUC (ng·h/mL)	1769 (28)	782 (35)	0.44 (0.38–0.51)
*t*_½_ (h)	8.0 (6.3–16.7)	20.0 (11.1–37.2)	–
*t*_max_ (h)	2.0 (2.0–4.0)	6.0 (4.0–12.0)	–
Metabolite:parent ratios
*C*_max_ ratio	0.32 (22)	0.01 (36)	–
AUC ratio	0.49 (24)	0.03 (33)	–

*Values are: geometric mean (% CV) for *C*_max_, AUC and metabolite:parent ratios; median (range) for *t*_½_, and *t*_max_. bid, twice daily; GLS, geometric least-square; CI, confidence interval; *C*_max_, maximum plasma concentration; AUC, area under the plasma concentration–time curve from time 0 to infinity; *t*_½_, terminal elimination half-life; *t*_max_, time to *C*_max_.

The AR-C124910XX plasma concentration–time profile was markedly decreased by co-administration of ketoconazole with ticagrelor (Figure 3b). Pharmacokinetic parameters for AR-C124910XX are summarized in Table 2. The half-life and *t*_max_ of AR-C124910XX were both prolonged in the presence of ketoconazole. The *C*_max_ and AUC for AR-C124910XX were decreased by 89% and 56%, respectively, by the co-administration of ketoconazole. The 90% CIs of the GLS mean ratio for both parameters were outside the limits of 0.70–1.43 (Table 2). Ketoconazole co-administered with ticagrelor markedly decreased the metabolite:parent ratios *C*_max_ and AUC by 97% and 94%, respectively (Table 2).

### Effect of ticagrelor on ketoconazole plasma concentrations

Co-administration of ticagrelor with ketoconazole did not affect the steady-state plasma concentrations of ketoconazole. The mean (% CV; range) plasma concentrations of ketoconazole pre-dosing on day 4 (i.e., in the absence of ticagrelor) were 1410 (73; 396–3540) ng/mL, and at 12 hours post-dosing with ticagrelor on day 4 were 1271 (84;334–4150) ng/mL.

### Safety and tolerability

Ticagrelor was well tolerated in the absence or presence of diltiazem. No AEs were reported following a single oral dose of ticagrelor. Overall, 14 mild AEs were reported in eight volunteers receiving ticagrelor + diltiazem, all of which resolved by the end of the study; 13 of these AEs were considered to be treatment related. Headache, the most common AE, was reported as a single AE in six volunteers and occurred four times in another volunteer. The other AEs were dizziness (*n* = 1), pharyngolaryngeal pain (*n* = 1), somnolence (*n* = 1), and pruritus (*n* = 1).

Co-administration of ticagrelor with ketoconazole was well tolerated. Overall, six volunteers had seven AEs during the co-administration of ticagrelor and ketoconazole, and four volunteers had five AEs with ticagrelor alone. Of these 12 AEs, seven were considered to be related to treatment. Eleven AEs were mild and resolved without intervention. One volunteer had a right bundle branch block which was present after the 14-day washout period following ticagrelor + ketoconazole co-administration, and discontinued the study; this event was considered mild and not to be treatment-related. Two bleeding-related AEs occurred with ticagrelor + ketoconazole: one event of hematoma and one event of hemorrhoidal hemorrhage. The other AEs in the ticagrelor + ketoconazole treatment were: flatulence, attention disturbance, headache, and dysmenorrhea. During administration of ticagrelor alone, the five AEs recorded in four volunteers were: flatulence, photophobia, viral conjunctivitis, increased appetite, and joint swelling.

No deaths or serious AEs occurred in either study. In both studies, no clinically meaningful changes in hematology, clinical chemistry, urinanalysis, vital signs (including blood pressure and pulse rate), physical findings, or ECG ketoconazole study data were observed. As expected with diltiazem, which has an approved indication for the treatment of hypertension, there was a marginal decrease in systolic and diastolic blood pressure compared to baseline. This decrease was not prolonged. Additionally, in the diltiazem study, two volunteers had abnormal ECG evaluations of first-degree atria-ventricular block during administration of diltiazem/ticagrelor, which resolved by the end of the study. This finding is consistent with the pharmacology of diltiazem.

## Discussion

CYP3A is abundant in the intestine and liver29, and is a key enzyme involved in the metabolism of many xenobiotics11,12. Extensive investigations have established that drugs can act as substrates, inhibitors, or inducers of CYP3A29. Consequently, clinically significant drug–drug interactions can occur which affect efficacy and/or safety9,10,12. *In vitro* assessments have demonstrated that ticagrelor is a substrate and a weak inhibitor of CYP3A18. Thus, co-administration of CYP3A inhibitors would be expected to increase exposure to ticagrelor and to reduce its metabolism to AR-C124910XX.

The results of the present studies using a moderate (diltiazem) and a strong (ketoconazole) CYP3A inhibitor confirm *in vitro* data that ticagrelor is a substrate for CYP3A. Pharmacokinetic parameters of ticagrelor and its primary metabolite, AR-C124910XX, were used to assess the effects of CYP3A inhibitors. The *C*_max_ and AUC values for ticagrelor and AR-C124910XX following a single oral dose of ticagrelor alone were consistent between the current interaction studies. Moreover, these results were also comparable with findings from studies in healthy volunteers14. For example, following a single oral dose of ticagrelor (100 mg) in healthy volunteers (*n* = 9), mean ± standard deviation (SD) for AUC was 3683 ± 753 and 1460 ± 408 ng·h/mL for ticagrelor and AR-C124910XX, respectively14. The consistency of pharmacokinetic parameters for ticagrelor and AR-C124910XX in the present studies with other results confirms their validity in investigating drug–drug interactions with model CYP3A inhibitors.

Our findings demonstrated that moderate inhibition of CYP3A by diltiazem resulted in a near doubling of ticagrelor’s half-life, and significantly increased *C*_max_ (69%) and AUC (174%) for ticagrelor. The metabolism of ticagrelor was also reduced as shown by the significant reduction in *C*_max_ and the slight decrease in AUC for AR-C124910XX, such that the metabolite:parent ratio was reduced from approximately 30% (without diltiazem) to 10% (with diltiazem). These results demonstrate that CYP3A is involved in the conversion of ticagrelor to AR-C124910XX and confirm the *in vitro* findings18.

It is well-recognized that there is a wide overlap of substrates between CYP3A and P-glycoprotein29, and studies have shown that ticagrelor is a substrate for both proteins18 (AstraZeneca, data on file). Given that diltiazem is also an inhibitor of P-glycoprotein10, its action in increasing ticagrelor exposure may result from inhibition of both CYP3A and P-glycoprotein.

Increased exposure to ticagrelor and reduced metabolism to its active metabolite (AR-C124910XX) resulting from diltiazem co-administration are unlikely to affect the efficacy of ticagrelor. Both the parent compound and AR-C124910XX directly inhibit platelet aggregation30 and are approximately equipotent in this respect (AstraZeneca, data on file). Therefore, the decreased exposure to AR-C124910XX may offset some of the effect of the increased exposure to the parent compound. However, as increased exposure to ticagrelor may result in prolonged inhibition of platelet aggregation (IPA), the incidence of bleeding events may also increase. No such events were reported in the ticagrelor-diltiazem study, albeit with a single 90-mg ticagrelor dose in healthy volunteers (who may not have the same propensity for bleeding as ACS patients).

Similar proportional increases in exposure to ticagrelor have been reported in patients with atherosclerosis receiving 200 mg ticagrelor twice daily (mean AUC 15,104 ng·h/mL, CV 39%) compared with those receiving 100 mg ticagrelor twice daily (5337 ng·h/mL, CV 45%), at 28 days, i.e. a 283% increase in exposure compared with a dose similar to the recommended dose of ticagrelor16. This almost 3-fold increase in exposure to ticagrelor was only associated with a slight increase in minor bleeding events, i.e. 17/39 (44%) and 19/37 (51%) of patients in the twice-daily 100 and 200 mg ticagrelor groups, respectively16. In patients with ACS, a similar 277% increase in ticagrelor exposure at week 4 was reported in those receiving 180 mg ticagrelor twice daily compared with 90 mg twice daily (mean AUC ± SD, 90 mg twice daily: 4762 ± 2443 ng·h/mL; 180 mg twice daily: 13,198 ± 4982 ng·h/mL), which was associated with a dose-dependent increase in IPA17. Total bleeding events in these patients with ACS were comparable between the two treatment groups: number of events (Kaplan–Meier event rates) 90 mg twice daily: 32 (9.8) and 180 mg twice daily: 25 (8.0) through week 4; and 90 mg twice daily: 34 (10.9) and 180 mg twice daily: 33 (11.4) through week 12^31^. Collectively, these data indicate that the magnitude of increase in ticagrelor exposure seen with a moderate CYP3A inhibitor is unlikely to result in significant bleeding events. Indeed, the use of moderate CYP3A inhibitors was permitted with ticagrelor in PLATO2,32. Ticagrelor can be co-administered with drugs classified as moderate CYP3A inhibitors, with no dose adjustment3,4.

Strong inhibition of CYP3A activity by ketoconazole co-administration markedly increased *C*_max_ (135%) and AUC (632%) of ticagrelor, versus ticagrelor alone. Furthermore, these parameters for AR-C124910XX were significantly decreased by 89% and 56%, respectively. As with diltiazem, ketoconazole is also an inhibitor of P-glycoprotein10. As discussed above, the action of ketoconazole in increasing ticagrelor exposure may result from inhibition of both CYP3A and P-glycoprotein. Further studies are needed to assess the respective contributions of these pathways to increased ticagrelor exposure.

Of the 14 healthy volunteers in the ticagrelor-ketoconazole study, two bleeding events were recorded, possibly due to the higher exposure to ticagrelor. In contrast, the maximum tolerated single dose of ticagrelor (900 mg) in healthy volunteers (*n* = 6) resulted in a much higher exposure to ticagrelor (mean [% CV], *C*_max_ 5153 [42] ng/mL; AUC 39,153 [38] ng·h/mL) and no bleeding-related AEs were recorded26,27. However, both of these healthy volunteer studies were of short duration and only investigated a single ticagrelor dose. As the co-administration of the strong CYP3A inhibitor, ketoconazole, resulted in a ≥5-fold increase in ticagrelor AUC, this finding indicates a significant drug–drug interaction10. Thus, long-term co-administration of ticagrelor with drugs classified as strong CYP3A inhibitors is not recommended3,4.

In addition to inhibiting CYP3A activity, diltiazem and ketoconazole are also substrates for CYP3A33,34. Ticagrelor has been shown to be a weak-to-moderate inhibitor of CYP3A activity *in vitro*18. However, in the current studies, a single dose of ticagrelor did not affect the pharmacokinetics of diltiazem or the ability to achieve steady-state ketoconazole plasma levels. In contrast, while the current data do not apparently support *in vitro* observations, other healthy volunteer studies have demonstrated that ticagrelor can have a clinically significant effect by increasing exposure to other CYP3A substrates, such as atorvastatin and simvastatin. A recent study demonstrated that ticagrelor increased mean atorvastatin *C*_max_ and AUC by 23% and 36%, respectively35. Similarly, simvastatin *C*_max_ and AUC were increased by 81% and 56% with ticagrelor35.

It should be noted that all volunteers in the diltiazem study were Hispanic, whereas the majority of volunteers in the ketoconazole study were Caucasian. As some differences in CYP3A activity have been observed between different races36, some caution should be used in applying these data more broadly.

Patients with ACS have a wide variety of co-morbidities and often receive multiple drugs7,8. The results of the present studies indicate that clinically significant drug–drug interactions with ticagrelor are likely to occur with co-administration of strong, but not moderate, CYP3A inhibitors. Examples of drugs which are strong CYP3A inhibitors include certain antifungal agents (e.g., itraconazole), antibiotics (e.g., clarithromycin, telithromycin), antiretroviral drugs (e.g., ritonavir, atazanavir) and antidepressants (e.g., nefazodone)10. Thus, co-administration of such agents is contraindicated with ticagrelor, and alternative drugs should be considered3,4. However, ticagrelor can be co-administered with moderate CYP3A inhibitors and no dose adjustment is required3,4.

## Conclusion

In conclusion, co-administration of ticagrelor with CYP3A inhibitors resulted in higher exposure to ticagrelor and lower exposure to its metabolite, AR-C124910XX. The magnitude of these effects suggests that moderate CYP3A inhibitors can be co-administered with ticagrelor without the need to modify the dose of ticagrelor. In contrast, co-administration of ticagrelor with strong CYP3A inhibitors is not recommended.
